# Socioeconomic inequality in mental well-being associated with COVID-19 containment measures in a low-incidence Asian globalized city

**DOI:** 10.1038/s41598-021-02342-8

**Published:** 2021-11-30

**Authors:** Roger Yat-Nork Chung, Gary Ka-Ki Chung, Siu-Ming Chan, Yat-Hang Chan, Hung Wong, Eng Kiong Yeoh, Jessica Allen, Jean Woo, Michael Marmot

**Affiliations:** 1 CUHK Institute of Health Equity, The Chinese University of Hong Kong, Hong Kong SAR, China; 2 The Jockey Club School of Public Health and Primary Care, The Chinese University of Hong Kong, Hong Kong SAR, China; 3 CUHK Institute of Ageing, The Chinese University of Hong Kong, Hong Kong SAR, China; 4 CityU Department of Social and Behavioural Sciences, The City University of Hong Kong, Hong Kong SAR, China; 5 Department of Social Work, The Chinese University of Hong Kong, Hong Kong SAR, China; 6 Department of Epidemiology and Public Health, UCL Institute of Health Equity, UCL Research, London, UK; 7 4/F, School of Public Health and Primary Care, Prince of Wales Hospital, Shatin, NT, Hong Kong SAR China

**Keywords:** Epidemiology, Quality of life

## Abstract

The COVID-19 pandemic exposes and amplifies pre-existing inequalities even in places with relatively well-controlled outbreaks such as Hong Kong. This study aimed to explore whether the socioeconomically disadvantaged fare worse via various types of worry in terms of their mental health and well-being. Between September and October 2020, 1067 adults in Hong Kong were recruited via a cross-sectional population-wide telephone survey. The inter-relationship between deprivation, types of worry, mental health disorders, and subjective well-being was assessed using structural equation modelling. Results showed significant total effects of deprivation on worries about being infected (p = 0.002), economic activities and livelihood (p < 0.001), and personal savings (p < 0.001), as well as mental health disorders (p < 0.001) and subjective well-being (p < 0.001). Specifically, worry about economic activities and livelihood partly mediated the total effect of deprivation on mental health disorders (p = 0.004), whereas worry about personal savings and worry about economic activities and livelihood partially mediated the total effect of deprivation on subjective well-being (p = 0.007 and 0.002, respectively). Socioeconomic inequality, particularly in mental health and well-being, could be exacerbated via people’s economic concerns during the pandemic, which was largely induced by the COVID-19 containment measures rather than the pandemic per se given the relatively low COVID-19 incidence in Hong Kong.

## Introduction

As of the end of September 2021, the coronavirus disease 2019 (COVID-19) has infected more than 230 million people and killed almost 4.8 million worldwide since the inception of the pandemic^1^. It has been suggested that the pandemic exposes and amplifies the underling inequalities in health^2,3^ and disproportionately affects the socially disadvantaged people of lower socioeconomic position^4,5^ and communities of color^6–8^. These patterns are more apparent for places with relatively higher incidence and mortality rates from COVID-19, but less apparent for places with relatively fewer cases. But even in places with relatively lower COVID-19 incidence and mortality rates such as Hong Kong, it has been found that social inequality in health, especially mental health, could still be attributed to the pandemic via people’s general worry over COVID-19 and impact on their economic activity status^9^.

In places where incidence and mortality rates are relatively higher, and where new variants of the virus are rampant, it is understandable that people would be more worried about themselves or others being infected with the potentially deadly virus or the prognosis when they are actually being infected. However, it is relatively less known in the literature as to the types of worry people might regard to the disease in places where the COVID-19 incidence and mortality rates are kept at a relatively low level. In some places, the relatively low incidence comes at a price of stringent or even draconian containment and mitigation measures, such as Hong Kong, where mandatory face-mask wearing, social distancing, occasional lockdown of schools, vendors and restaurants, partial border closure, mandatory testing for certain service industries and residential buildings being affected have been in place since the earlier days of the pandemic^10^. As mentioned earlier, the people of lower socioeconomic position had worse mental health in Hong Kong during the pandemic due to the stringent public health measures that affect their economic activity status^9^. Therefore, we would want to further examine in the context of relatively low disease incidence and stringent measures (1) whether the types of worry vary across socioeconomic position, and (2) whether the socioeconomically disadvantaged fare worse in terms of their mental health and well-being via the different types of worry.

Hong Kong, a densely populated globalized Asian city located in Southern China with around 7.5 million people^11^, can be an exemplar setting to address the above questions, since it has a huge wealth inequality that reached an all-time high in 2016 with a Gini coefficient of 0.539^12^, a relatively low incidence and mortality of COVID-19 (i.e., 12,223 cases and 213 deaths as of the end of September 2021) as compared with the rest of the world, and stringent containment measures that impacted on the socioeconomic aspects of the whole population^13,14^. By focusing on a study setting with lower COVID-19 incidence and mortality, we would be able to distinguish the impact of the virus itself and the impact of the containment measures on people’s health more effectively. This distinction would be much more difficult to achieve in places being harder hit by the pandemic, where much higher proportions of the population are acquainted with people who had contracted the disease, and therefore would have more types of COVID-19-related worry and concern specific to having the disease, including but not limited to worry over the prognosis as well as the related medical expenses. Therefore, by addressing the two research questions raised above, we would also be able to distinguish and highlight the potential impact of the COVID-19-related containment measures on people’s health by their socioeconomic position.

## Subjects and methods

Data were collected from a population-wide random sample of households in Hong Kong via telephone survey from 11 September to 12 October, 2020. The inclusion criteria for the study were Hong Kong Chinese residents aged 18 or above. Upon successful contact with a target household, one qualified member of the household was selected among those family members using the last-birthday random selection method. Telephone interviews were carried out by experienced interviewers between 18:00 and 22:00 on weekdays. Prior appointments were arranged for suitable respondents in other periods including weekends and public holidays. Selected respondents with insufficient cognitive functioning for survey completion would be deemed ineligible, and that household would be excluded from this study. Among the 12,443 dialed telephone numbers, 10,555 were invalid cases in which 254 were non-residential lines, 4776 were fax lines/invalid lines, 1308 were cut off immediately, and 4217 were non-contacts after three attempts. Among the 1888 answered calls, 28 had mid-way termination, 59 could not establish contact with an eligible person after three attempts, and 734 were refused by the eligible persons, resulting in a final sample of 1067 respondents with a response rate of 56.5%. After excluding 14 respondents with missing data on education level, 1053 respondents were included in this study for analyses.

As our respondents were older and had a higher proportion of female compared with the general Hong Kong population, proportional weighting was adopted to ensure the representativeness of our surveyed adults for the general population with respect to the age and sex distribution in mid-2020 obtained from the Census and Statistics Department of the Hong Kong Government (Supplementary Table 1). All data analyses in this study were based on the weighted sample.

This study has been approved by the Joint Chinese University of Hong Kong – New Territories East Cluster Clinical Research Ethics Committee in July 2020.

## Measurements

Information on respondents’ deprivation status, worries related to COVID-19, mental health disorders, subjective well-being, and other socio-demographic and health factors were collected during the COVID-19 outbreak in Hong Kong, with details below.

### Material and social deprivation

The deprivation status of respondents was assessed and adopted as the primary socioeconomic indicator in this study. Following Townsend’s theory of relative deprivation which has been defined as a lack of command over resources covering material and social necessities^15^, a 21-item Deprivation Index (DI) was used to assess whether respondents could (not) afford a range of items that were considered as necessities by the majority of adults in the context of Hong Kong^15^. The 21 items were grouped into measures of social deprivation (4 items) and material deprivation (17 items). Specifically, measures of material deprivation cover items on food deprivation, clothing, medical care deprivation, household facilities and equipment, repair and maintenance, and finance. The DI has a good reliability with a Cronbach’s alpha of 0.832. A higher DI score represents a greater level of material and social deprivation, whereas a DI score of 2 or above was considered “deprived” for descriptive analyses^15^. The DI has been widely used in previous local studies which consistently supported the independent effect of deprivation on various health outcomes compared with other conventional socioeconomic indicators, including income-defined poverty^9,16–19^. Further details on the construction, validity and reliability of the DI had been described previously^15^.

### Worries related to COVID-19

Respondents were asked whether they had any of the following four types of worry related to COVID-19: (i) worry about being infected, which applied to both the respondents themselves and their family members; (ii) worry about economic activities and livelihood, in terms of job loss, salary reduction, and working time reduction; (iii) worry about supply of personal protective equipment (PPE), including face masks, hand sanitizers, and other hygiene products; and (iv) worry about their personal savings. The extent of worries was assessed in five levels: (i) very worried; (ii) worried; (iii) neutral; (iv) not worried; and (v) not worried at all.

### Mental health disorders

The 4-item Patient Health Questionnaire for Depression and Anxiety (PHQ-4)^20^, which combines the Patient Health Questionnaire-2 (PHQ-2) and the Generalized Anxiety Disorder-2 (GAD-2) scales, was adopted to assess the depression and anxiety symptoms of respondents during COVID-19. In each of the four items on the frequency of experiencing certain depression and anxiety symptoms, respondents were scored as 0 for “not at all,” 1 for “several days,” 2 for “more than half the days,” and 3 for “nearly every day.” A score of 3 or above was considered having at least mild symptoms of depression and anxiety for descriptive analyses in this study. All the four items served as the observed variables for the latent construct of “mental health disorders” in the structural equation modelling (SEM) analyses.

### Subjective well-being

The subjective well-being of respondents was measured with reference to the guidelines of the Organisation for Economic Co-operation and Development (OECD) for measuring subjective well-being^21^. Respondents were asked to rate themselves on a scale from 0 to 10 for each of the following three questions: (i) Overall, how satisfied are you with life as a whole these days? (2) Overall, to what extent do you feel the things you do in your life are worthwhile? and (3) How happy you felt yesterday? The higher the respective scores, the better the subjective well-being our respondents have. All the three items served as the observed variables for the latent construct on “subjective well-being” in the SEM analyses.

### Potential confounding

Information of age, sex, educational level, social security status, and number of chronic diseases were collected for confounding control. Specifically, education level was classified in to five groups: (i) primary level or below; (ii) lower secondary level; (iii) upper secondary level; (iv) sub-degree level (e.g., diploma programmes, certificate courses); and (v) degree level or above. Anyone receiving the means-tested Comprehensive Social Security Assistance (CSSA) Scheme provided by the Hong Kong Government would be regarded as a recipient of social security. Also, by assessing the chronic conditions or disability with which the respondents had ever been diagnosed by a medical doctor, the number of chronic diseases was derived.

## Statistical methods

Descriptive statistics of respondents by deprivation status were derived. Continuous variables are presented as mean with standard deviations (SD) and categorical variables as count numbers with percentages. Independent two-sample t-tests (for continuous variables) and Mantel–Haenszel test for trend (for categorical variables) were used to test the statistical differences in respondents’ characteristics between the deprived and the non-deprived.

The inter-relationship among deprivation score, level of worries related to COVID-19, mental health disorders, and subjective well-being were tested via SEM, with adjustments for age, sex, education level, social security status, and number of chronic diseases. SEM is a statistical methodology to examine the plausibility of theoretical models that attempt to explain relationships among variables^22^. SEM can analyse multiple independent variables, dependent variables, and their corresponding error terms within a theoretical framework while taking measurement errors into account. It can also address the mediating and moderating effects by model estimation among variables with interconnections^23^. A covariance matrix among all the aforementioned variables was generated for SEM analyses. Confirmatory factor analysis and reliability test were performed for the two latent constructs, i.e., mental health disorders and subjective well-being, to ensure that the latent constructs are well-explained by the corresponding observed variables with acceptable reliability. Observed variables with factor loadings above 0.50 represent a satisfactory measurement model^24^, whereas a Cronbach’s alpha above 0.80 indicates a good reliability^25^.

We obtained the regression weights of variables, the direct and indirect effects on the endogenous variables, and the goodness-of-fit of model from the SEM model. As the sample size in this study was relatively large (> 200), the checking of χ^2^ and its p-value can be overlooked^26^. SEM model with a root mean square error of approximation (RMSEA) value below 0.08 is deemed having a good model fit^22^. Other goodness-of-fit indices, including comparative fit index (CFI), incremental fit index (IFI), and Tucker-Lewis index (TLI), are considered to be satisfactory if they are higher than 0.90^27^ or considered to be superior if it is above 0.95^22^. The adjusted GFI (AGFI) are considered acceptable if the value is above 0.90. SPSS and AMOS version 26 were employed for statistical analyses. All statistical tests were two-tailed with a significant level of 0.05.

## Results

Table 1 shows the descriptive statistics of our weighted sample. 8.9% of our respondents were deprived. Slightly more than half (55.2%) of the respondents were female, 33.2% were 18–39 years, 45.6% were 40–64 years, and 21.2% were 65 years or above. Regarding types of worries due to COVID-19, 50.8% respondents were worried about being infected, 44.6% were worried about economic activities and livelihood, 3.0% were worried about the supply of PPE, and 34.2% were worried about personal savings. In terms of mental health disorders, 10.4% respondents had depression and anxiety symptoms. As for subjective well-being, the corresponding weighted mean scores of happiness, worthiness of life, and life satisfaction were 6.64 (SD = 1.57), 7.08 (SD = 1.56), and 6.48 (SD = 1.65). Between the deprived and the non-deprived, statistically significant differences were observed in most observed variables except sex and life satisfaction, with worry about being infected and happiness having marginal significant differences.

**Table 1:** Basic characteristics of the weighted sample by deprivation status (N = 1053).

	Total	Deprivation status		*p*-value^a^
		Non-deprived (91.1%)	Deprived (8.9%)	
	Weighted % or Mean ± SD	Weighted % or Mean ± SD	Weighted % or Mean ± SD	
**Age**				< 0.001
18–39 years	33.2	35.5	9.6	
40–64 years	45.6	44.5	56.4	
65 years or above	21.2	20.0	34.0	
**Sex**				0.268
Male	44.8	45.3	39.4	
Female	55.2	54.7	60.6	
**Education level**				< 0.001
Primary or below	17.2	14.8	41.5	
Lower secondary	12.9	11.5	27.7	
Upper secondary	31.3	32.2	22.3	
Sub-degree	8.8	9.3	4.3	
Degree or above	29.7	32.2	4.3	
**Social security status**				< 0.001
Yes	2.3	1.0	15.1	
No	97.7	99.0	84.9	
**Number of chronic diseases**				< 0.001
0	71.6	73.2	54.8	
1	20.2	19.8	24.7	
2	6.4	5.6	14.0	
3 or above	1.8	1.4	6.5	
**Types of worry**				
***Worry about being infected***				0.064
Not worried at all	2.8	2.8	2.1	
Not worried	36.2	36.1	37.2	
Neutral	10.2	10.7	5.3	
Worried	35.5	36.7	23.4	
Very worried	15.3	13.6	31.9	
***Worry about economic activities and livelihood***				< 0.001
Not worried at all	4.2	4.3	3.2	
Not worried	40.2	42.1	20.4	
Neutral	11.0	11.1	9.7	
Worried	29.8	29.6	32.3	
Very worried	14.8	12.9	34.4	
***Worry about supply of personal protective equipment***				0.034
Not worried at all	19.3	18.3	29.0	
Not worried	74.1	76.4	50.5	
Neutral	3.6	2.7	12.9	
Worried	1.6	1.7	1.1	
Very worried	1.4	0.9	6.5	
***Worry about personal savings***				< 0.001
Not worried at all	4.7	4.8	4.3	
Not worried	45.0	48.1	13.8	
Neutral	16.0	16.0	17.0	
Worried	24.6	23.5	36.2	
Very worried	9.6	7.7	28.7	
**Depression and anxiety symptoms**				< 0.001
None	89.6	90.9	75.3	
Mild/moderate/severe	10.4	9.1	24.7	
**Happiness (out of 10)**	6.64 ± 1.57	6.66 ± 1.56	6.36 ± 1.60	0.072
**Worthiness of life (out of 10)**	7.08 ± 1.56	7.16 ± 1.55	6.25 ± 1.46	< 0.001
**Satisfaction towards life (out of 10)**	6.48 ± 1.65	6.50 ± 1.65	6.23 ± 1.65	0.128

^a^Mantel-Haenszel test for trend was used for categorical variables while independent-sample t-test was used for continuous variables

The covariance matrix among all observed variables used in the SEM model is displayed in Table 2, whereas the standardized factor loadings of observed variables on the two latent constructs in confirmatory factor analysis are presented in Table 3. The corresponding factor loadings ranged from 0.729 to 0.855 for mental health disorders, and from 0.570 to 0.948 for subjective well-being. High reliability was observed for the two latent constructs (Cronbach’s alpha = 0.888 and 0.807, respectively).

**Table 2:** Covariance matrix of observed variables on the weighted sample (N = 1053).

	1	2	3	4	5	6	7	8	9	10	11	12	13	14	15	16	17
1. Deprivation score	1.533																
2. Worry about being infected	0.136	1.378															
3. Worry about economic activities and livelihood	0.329	0.609	1.446														
4. Worry about supply of personal protective equipment	0.048	0.031	0.128	0.418													
5. Worry about personal savings	0.405	0.428	1.024	0.163	1.26												
6. Depression symptoms (1st item of PHQ-2)	0.122	0.025	0.093	−0.012	0.069	0.223											
7. Depression symptoms (2nd item of PHQ-2)	0.154	0.03	0.121	−0.015	0.094	0.171	0.234										
8. Anxiety symptoms (1st item of GAD-2)	0.137	0.035	0.098	−0.022	0.099	0.145	0.15	0.263									
9. Anxiety symptoms (2nd item of GAD-2)	0.178	0.029	0.102	0.006	0.116	0.151	0.155	0.153	0.206								
10. Happiness	−0.242	−0.116	−0.478	0.042	−0.476	−0.339	−0.317	−0.345	−0.274	2.45							
11. Worthiness of life	−0.389	−0.166	−0.342	0.032	−0.357	−0.235	−0.249	−0.206	−0.257	1.321	2.438						
12. Life satisfaction	−0.221	−0.286	−0.686	0.047	−0.498	−0.29	−0.288	−0.288	−0.221	1.944	1.167	2.724					
13. Age	0.564	−0.56	−0.432	0.178	−0.147	−0.205	−0.158	−0.245	−0.145	1.287	0.26	1.868	12.576				
14. Sex	0.009	0.089	0.012	0.011	0.004	0.004	−0.003	0.016	0.005	0.047	0.019	0.051	−0.023	0.247			
15. Social security status	−0.044	0.001	−0.001	−0.006	−0.009	0.003	0.002	0.003	0.001	−0.007	0.009	−0.009	−0.083	−0.002	0.022		
16. Education level	−0.395	−0.033	−0.104	−0.087	−0.179	0.058	0.023	0.065	0.037	−0.383	0.04	−0.49	−3.353	−0.08	0.035	2.05	
17. Number of chronic diseases	0.073	−0.015	−0.032	0.028	−0.021	0.001	0.008	−0.022	−0.002	0.153	−0.016	0.201	1.291	−0.014	−0.021	−0.419	0.491

**Table 3:** Standardized factor loadings of observed variables on constructs.

Latent construct	Observed variables	Factor loading
Mental health disorders	1. Little interest or pleasure in doing things	0.852
(Cronbach’s alpha = 0.888)	2. Feeling down, depressed, or hopeless	0.840
	3. Feeling nervous, anxious or on edge	0.729
	4. Not being able to stop or control worrying	0.855
Subjective well-being	1. Happiness	0.794
(Cronbach’s alpha = 0.807)	2. Worthiness of life	0.570
	3. Life satisfaction	0.948

**Figure 1:**
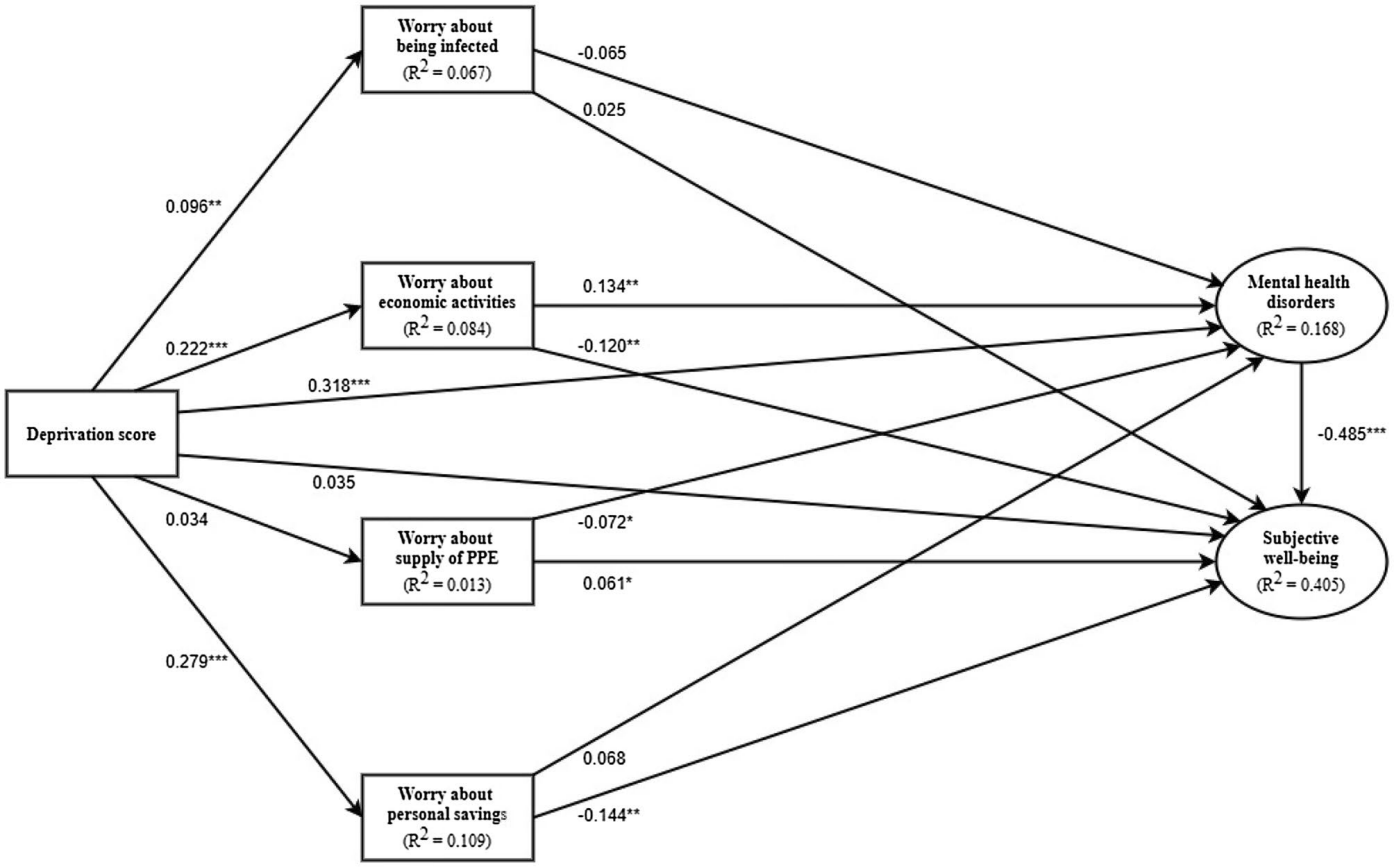
SEM results on the associations among deprivation, types of worry, mental health disorders and subjective well-being. Note: The SEM model was adjusted for age, sex, education level, social security status and number of chronic diseases. *p < 0.05; **p < 0.01; ***p < 0.001.

The resultant SEM model yielded good model fit to the data, with χ^2^ (df = 64, N = 1053) = 345.510, *p* < 0.001, RMSEA = 0.065, RMR = 0.079 CFI = 0.960, IFI = 0.960, TLI = 0.914, AGFI = 0.911. With adjustments for age, sex, education level, social security status, and number of chronic diseases in the SEM model, the total effects of deprivation on mental health disorders (*β* = 0.358; *p* < 0.001) and subjective well-being (*β* = −0.201; *p* < 0.001) were statistically significant. As shown in Fig. 1, significant direct effects of deprivation were observed on worry about being infected (*β* = 0.096; *p* = 0.002), worry about economic activities and livelihood (*β* = 0.222; *p* < 0.001), worry about personal savings (*β* = 0.279; *p* < 0.001), and mental health disorders (*β* = 0.318; *p* < 0.001). There was a significant direct effect of worry about economic activities and livelihood on mental health disorders (*β* = 0.134; *p* = 0.007), and it partly mediated the association between deprivation and mental health disorders (*β* = 0.222 × 0.134 = 0.030; *p* = 0.004). On the other hand, despite the significant total effect of deprivation on subjective well-being, the observed non-significant direct effect (*β* = 0.035; *p* = 0.271) indicated that deprivation was only associated with subjective well-being through indirect mediation. As worry about economic activities and livelihood as well as worry about personal savings exhibited significant direct effects on subjective well-being (*β* = −0.120; *p* = 0.009 and *β* = −0.144; *p* = 0.003, respectively), both worries partially mediated the association between deprivation and subjective well-being (*β* = 0.222 x −0.120 = −0.027; *p* = 0.007 and *β* = 0.279 x −0.144 = −0.040; *p* = 0.002, respectively). In addition, mental health disorders exerted strong direct effect on subjective well-being (*β* = −0.485; *p* < 0.001), and substantially mediated the association between deprivation and subjective well-being (*β* = −0.154; *p* < 0.001). Overall, the SEM model explained 16.8% of the variance of mental health disorders and 40.5% of the variance of subjective well-being.

## Discussion

### Deprivation and types of worry

The SEM showed that deprivation was most strongly associated with worry about personal savings, followed by worry about economic activities and livelihood, with worry about being infected trailing behind; however, deprivation was found not to be significantly associated with worry about the supply of PPE. This finding echoes with studies in other settings, where one study found that low-income and economically inactive individuals were more prone to loneliness during the pandemic in the UK^28^, and the other one in China found that individuals with higher socioeconomic status have richer resources in coping with COVID-19 and are thus less worried^29^. On the other hand, there are consistency and apparent inconsistency with the findings of our previous Hong Kong study from an earlier phase of the pandemic (i.e., Spring 2020)^9^, which found that being deprived were more associated with job loss and instability, greater worry of the pandemic, as well as low reserve of face masks in the household than being non-deprived. The comparison of this earlier local research with the present study could imply that while the concern over job loss and instability as well as the pandemic in general were persistent from early to late 2020, supply of PPE became less of a concern even for the deprived who initially tended to have inadequate reserve in their households. This is reasonable because the price of face masks was significantly ramped up due to a general shortage in the market in the earlier days of the pandemic^30^, and they had since become increasingly available and affordable, possibly due to market saturation as well as civil society and social mobilization in the community^31^.

### Types of worry as mediator

Our study made further distinction in terms of the types of worry that mediates the association between deprivation and health outcomes. From our mediation analysis, we found that only two types of worry could significantly mediate the association of deprivation with the mental health and well-being of our respondents. In particular, worry about economic activities and livelihood was a significant mediator of the association between deprivation and mental health disorders as well as the association between deprivation and subjective well-being, while worry about personal savings was a significant mediator of the association between deprivation and subjective well-being. These findings are consistent with those from other settings with greater COVID-19 incidence including the US, the UK, and also some developing countries, where it was found that worsening mental health was most strongly associated with concerns about the economic consequences of the pandemic^32,33^, income loss and financial strain were uniquely associated with depressive symptoms and their exacerbation over time, above and beyond pandemic-related anxiety^34^, and reduced work and income were associated with decreased psychological well-being and increased anxiety, depression, and loneliness^35^. Our present findings is also consistent with the findings from our previous study, where being deprived was found to have a significant indirect effect on mental health with worry about the pandemic in general and actual job loss/instability as the mediating variables^9^. In particular, we also found that worry about being infected or about supply of PPE did not mediate the associations between deprivation and health outcomes. In other words, even though there were genuinely more concern over being infected among the deprived as mentioned earlier, this type of worry might not affect people’s mental health or well-being. This is a reasonable finding given that Hong Kong’s COVID-19 disease incidence has been relatively low compared with other countries in the world, and that the supply of PPE has become less of a concern even for the deprived as PPE became increasingly available and affordable in the market, as mentioned earlier.

### The pervasive mental health inequality related to deprivation

Finally, there was a significant effect from deprivation to mental health disorder that was direct and unmediated by the various worries over the pandemic. This is something not to be overlooked because it implies that deprivation could lead to worse mental health outcomes not only via worries over the various aspects of the pandemic. There has been ample amount of solid evidence over the past decades that confirmed the association between deprivation and mental health outcomes even during the pre-COVID-19 period^19,36–38^. However, it has been suggested that this pervasive, underlying health inequality could be further exposed and amplified by the destructive pandemic^2,3,39^.

### Public health implications

Taken together, our findings confirmed that even in a city where the COVID-19 incidence was relatively low in the world, socioeconomic inequality in terms of mental health outcomes and well-being did exist, and could be partially mediated by COVID-19-related economic-specific concerns. These findings send an important message to the public and the policymakers at large that people’s health and well-being could be affected not only by being infected with the viral disease, but by genuine economic concerns during the pandemic that were related to the containment measures. In fact, it has been found that biological disaster of COVID-19 and other similar epidemics such as SARS not only have strong impact on the mental health of those being infected and their acquaintances, but also the well-being of the general public^40^. While it is indisputable that the containment measures were meant to achieve the public health functions of disease prevention and health protection, policymakers should bear in mind that these measures could also negatively affect our health via other social determinants of health. Therefore, containment policies need to be devised and amended if necessary to take the social determinants of health into account. For instance, containment measures that disproportionately affect the economic activities of certain groups of people (e.g., people of lower socioeconomic position or of ethnic minority and migrant background) need to be accompanied by other corresponding policies that address and alleviate the economic burden of the ones being affected^41^.

### Limitations

There are several caveats to our study. First, this was cross-sectional in design; therefore, we need to be cautious in inferring any causal relationships between the predictors and the outcomes. Second, there may be sampling bias in terms of the telephone survey design, since those who did not own a landline phone number may be systematically different from those who did^42^. Also, those who did not answer, cut the phone call upon ringing, or dropped out in the middle of the interview might also be different from those who spent time and effort to complete the survey; nevertheless, we are primarily interested in studying the associations among the variables in this study. Third, answers were self-reported, and therefore may incur recall bias in the results, which is inherent in any survey that requires self-reporting; and since telephone interviewers are generally tight on time, the respondents’ answers could also be affected. Nevertheless, we made attempt to control for the potential recall and reporting bias during the study design stage by specifying a clear timeframe for the questions, and making amendments according to an initial pre-run pilot. Last, there may be other COVID-19-related aspects of worry that were not accounted for in our survey, including but not limited to changes in social and family relationship during time of social distancing^28,43^, social support and capital^29,44,45^, health behaviours^46,47^, and poorer healthcare access^48–50^. In fact, the list of unexplained variables could go on; nevertheless, our SEM could explain up to 40.5% of the variance of the observed associations with subjective well-being.

## Conclusion

While deprivation itself may directly lead to worse mental health, which could then affect people’s well-being, it could also lead to worse mental health via genuine economic concerns during a global pandemic. Socioeconomic inequality, particularly in mental health and well-being, can still be exacerbated via people’s economic concern during the pandemic even when the disease incidence was not high. Containment measures that severely affect people’s economic activities, livelihood, and thus personal savings must be accompanied by other corresponding policies that take the social determinants of health into account for economic alleviation purpose. The public health policies and the corresponding economic alleviation policies should be considered to have equal footing in terms of their degree of essentiality. Principle of equity requires action to be taken now.

### Ethics approval and consent to participate

This study was approved by Joint Chinese University of Hong Kong-New Territories East Cluster Clinical Research Ethics Committee in August 2020 (CREC Reference number. CRE-2020.378). All methods were carried out in accordance with relevant guidelines and regulations. Informed consent was obtained from all participants or, if participants are under 16, from a parent and/or legal guardian.

## Supplementary Information

Supplementary Information.

## Data Availability

The datasets used and/or analysed during the current study are available from the corresponding author on reasonable request.
