# Supplementary material for: Socioeconomic inequality in mental well-being associated with COVID-19 containment measures in a low-incidence Asian globalized city
Source: Sci Rep. 2021 Nov 30;11:23161. doi: 10.1038/s41598-021-02342-8 (PMC8633192; doi:10.1038/s41598-021-02342-8)
Supplement: Supplementary file 1 — Supplementary Information. [file 41598_2021_2342_MOESM1_ESM.docx]

**Socioeconomic inequality in mental well-being associated with COVID-19 containment measures in a low-incidence Asian globalized city**

Roger Yat-Nork CHUNG^1,2,3,†,^*, Gary Ka-Ki CHUNG^1,†^, Siu-Ming CHAN^1,4^, Yat-Hang CHAN^1^, Hung WONG^1,5^, Eng Kiong YEOH^1,2^, Jessica ALLEN^6^, Jean WOO^1,3^, Michael MARMOT^1,6^

^1^ CUHK Institute of Health Equity, The Chinese University of Hong Kong, Hong Kong SAR, China

^2^ The Jockey Club School of Public Health and Primary Care, The Chinese University of Hong Kong, Hong Kong SAR, China

^3^ CUHK Institute of Ageing, The Chinese University of Hong Kong, Hong Kong SAR, China

^4^ CityU Department of Social and Behavioural Sciences, The City University of Hong Kong, China

^5^ Department of Social Work, The Chinese University of Hong Kong, Hong Kong SAR, China

^6^ UCL Institute of Health Equity, UCL Research Department of Epidemiology and Public Health, London, UK

^†^ Equal contributions

* Correspondence to: Roger Yat-Nork CHUNG

4/F, School of Public Health and Primary Care, Prince of Wales Hospital, Shatin, NT, Hong Kong SAR, China

Email: [rychung@cuhk.edu.hk](mailto:rychung@cuhk.edu.hk)

Tel. no.: (852) 2252 8799

Fax no.: (852) 2606 3791

| **Supplementary Table 1. Weighting factors based on the distribution of age and sex of the mid-2020 Hong Kong Population** | | | | |
| --- | --- | --- | --- | --- |
| Sex | Age | Mid-2020 Hong Kong Population (N=6478200) | Sampled respondents  (N=1503) | Weighting factor |
|  |  | N (%) | N (%) |  |
| ***Male*** |  |  |  |  |
|  | 18-19 | 61100 (0.9) | 15 (1.4) | 0.662 |
|  | 20 - 24 | 189600 (2.9) | 19 (1.8) | 1.622 |
|  | 25 - 29 | 224900 (3.5) | 12 (1.1) | 3.046 |
|  | 30 - 34 | 230800 (3.6) | 13 (1.2) | 2.886 |
|  | 35 - 39 | 238900 (3.7) | 15 (1.4) | 2.589 |
|  | 40 - 44 | 227600 (3.5) | 16 (1.5) | 2.312 |
|  | 45 - 49 | 242700 (3.7) | 20 (1.9) | 1.972 |
|  | 50 - 54 | 241800 (3.7) | 28 (2.7) | 1.404 |
|  | 55 - 59 | 302500 (4.7) | 63 (6.0) | 0.780 |
|  | 60 - 64 | 293800 (4.5) | 18 (1.7) | 2.653 |
|  | 65 - 69 | 224600 (3.5) | 11 (1.0) | 3.319 |
|  | 70 - 74 | 165800 (2.6) | 28 (2.7) | 0.962 |
|  | 75 - 79 | 95100 (1.5) | 21 (2.0) | 0.736 |
|  | 80 - 84 | 80300 (1.2) | 22 (2.1) | 0.593 |
|  | ≥ 85 | 79200 (1.2) | 19 (1.8) | 0.678 |
| ***Female*** |  |  |  |  |
|  | 18-19 | 57000 (0.9) | 9 (0.9) | 1.029 |
|  | 20 - 24 | 190200 (2.9) | 27 (2.6) | 1.145 |
|  | 25 - 29 | 257600 (4.0) | 22 (2.1) | 1.903 |
|  | 30 - 34 | 321800 (5.0) | 31 (2.9) | 1.687 |
|  | 35 - 39 | 371400 (5.7) | 39 (3.7) | 1.548 |
|  | 40 - 44 | 345600 (5.3) | 45 (4.3) | 1.248 |
|  | 45 - 49 | 341700 (5.3) | 52 (4.9) | 1.068 |
|  | 50 - 54 | 316500 (4.9) | 86 (8.2) | 0.598 |
|  | 55 - 59 | 345000 (5.3) | 165 (15.7) | 0.340 |
|  | 60 - 64 | 301100 (4.6) | 46 (4.4) | 1.064 |
|  | 65 - 69 | 231400 (3.6) | 47 (4.5) | 0.800 |
|  | 70 - 74 | 172300 (2.7) | 50 (4.7) | 0.560 |
|  | 75 - 79 | 95100 (1.5) | 42 (4.0) | 0.368 |
|  | 80 - 84 | 94200 (1.5) | 48 (4.6) | 0.319 |
|  | ≥ 85 | 138600 (2.1) | 24 (2.3) | 0.939 |
